# Correlation of staging and risk factors with cardiovascular autonomic neuropathy in patients with type II diabetes mellitus

**DOI:** 10.1038/s41598-021-80962-w

**Published:** 2021-02-11

**Authors:** Muhanad M. Dhumad, Farqad B. Hamdan, Mahmood S. Khudhair, Hisham Y. Al-Matubsi

**Affiliations:** 1 Section of Pharmacy, Baghdad College of Medical Sciences, Baghdad, Iraq; 2 Department of Physiology, College of Medicine, Al-Nahrain University, Baghdad, Iraq; 3 Endocrinology Section, Department of Medicine, College of Medicine, Al-Nahrain University, Baghdad, Iraq; 4 Department of Pharmacology and Medical Sciences, University of Petra, Amman, Jordan

**Keywords:** Neuroscience, Physiology, Cardiology, Endocrinology, Medical research, Risk factors

## Abstract

The impairment of cardiovascular autonomic control among the underdiagnosed complication of diabetes mellitus (DM) with a high prevalence rate of up to 60% in type 2 DM (T2DM). Cardiac autonomic neuropathy (CAN) is an independent risk factor for cardiovascular mortality, arrhythmia, silent ischemia, any major cardiovascular event, and heart failure. We aimed to evaluate cardiovascular autonomic activity by different physiological maneuvers, study risk factors for diabetic CAN including age, gender, duration of diabetes, body mass index (BMI), and glycemic control, and correlate CAN stage with risk factors. One hundred and forty-two T2DM patients consisted of 62 males and 80 females and 100 volunteers as a control sample. Cardiac autonomic functions were assessed by Ewing's tests. Glycated hemoglobin (HbA1c), body weight, height, body mass index (BMI), and waist-hip ratio (WHR) were also measured. Cardiovascular autonomic functions and Ewing scores were significantly different in people with diabetes when compared with control healthy subjects. Ewings test values and Ewing scores were significantly different between diabetics with and without CAN and within patients with different CAN staging. People with diabetes with CAN have a significantly longer duration of disease when compared to those without CAN. A strong association has been found between CAN severity and patient age, duration of disease, HbA1c severity, and the WHR (*P* < 0.001) but not with BMI. The duration of disease and HbA1c level appear to be associated with the development of CAN (*P* = 0.001 and *P* = 0.008, respectively). The poorer glycemic control and the longer the duration of the disease, the higher the prevalence of CAN in T2DM. Age, duration of disease, WHR, and HbA1c are well correlated with the severity of CAN. Parasympathetic impairment is more sensitive to the detection of autonomic dysfunctions than do sympathetic impairment.

## Introduction

Diabetes mellitus (DM) is a chronic metabolic syndrome characterized by the presence of hyperglycemia^1^. Chronic hyperglycemia is associated with long-term microvascular complications manifested as retinopathy, nephropathy, and neuropathy^2^. Diabetic neuropathy includes peripheral neuropathy and autonomic neuropathy^3^. Disease duration and level of glycemic control are amongst the important causative factors which donated to the development of autonomic neuropathy (AN) in T2DM patients^4^.

In T2DM, AN is still an underdiagnosed cause of morbidity and mortality, particularly when it involves the cardiac functions in terms of cardiac autonomic neuropathy (CAN). This CAN result in cardiovascular dysfunction which is accompanied by the progression to myocardial ischemia, coronary artery disease, and stroke^5,6^.

CAN prevalence increases substantially with diabetes duration in T2DM (up to 60% after 15 years)^7^. Besides, CAN is present in some patients with prediabetes, insulin resistance, or metabolic syndrome^8^. Moreover, data validates an association between CAN and glucose variability, specifically in the hypoglycemic category^9^.

In T2DM, assessment of CAN symptoms and signs should be accomplished at the time of diagnosis, particularly for those with poor glycemic control (HbA1c > 7%), presence of major cardiovascular disease risk factor, or additional chronic complications of DM^4,6^.

We aimed to evaluate the autonomic cardiovascular function in patients with T2DM, the potential correlation between CAN and risk factors, and to associate CAN staging with risk factors.

## Methods

A cross-sectional study conducted from December 2018 to August 2019 in the College of Medicine, Al-Nahrain University, and Al-Imamian Al-Kadhmiyain Medical City, Baghdad, Iraq. The study was approved by the Institutional Review Board (IBR) of the College of Medicine, Al-Nahrain University (Decision number: mmm/155: Date: 11/12/2018), and informed consent was obtained from all the participants. All participants were reminded that their participation was voluntary, and they had the right to withdraw from the research at any stage.

### Subjects

One hundred and forty-two patients with T2DM who attended a diabetic clinic of 62 males and 80 females aged 41 to 68 years (53.35 ± 7.83 years) were studied. The duration of their illness ranges from 1 to 15 years [(5.39 ± 4.75 years), median = 6 years, interquartile range = 8 years]. Diagnosed cases of T2DM included in the study were those with symptoms of diabetes plus a random blood glucose concentration ≥ 200 mg/dL, fasting plasma glucose ≥ 126 mg/dL, and 2-h plasma glucose ≥ 200 mg/dL during the oral glucose tolerance test^10^.

People with diabetes with uncontrolled hypertension, ischemic heart disease, valvular heart disease, arrhythmia, including atrial fibrillation heart failure, severe illness such as malignancy and severe infection, liver cirrhosis, gestational diabetes, severe hypoglycemia, alcoholism, and those on beta-blocker medication were excluded from the study.

Another one hundred age- and sex-matched healthy subjects composed of 39 males and 61 females serve as the control group. Their age ranges from 35 to 65 years (51.73 ± 8.81 years).

### Medical history and examination

A detailed medical history and clinical examination were done by senior Diabetologist. Bodyweight, height, BMI, and WHR have been recorded. Fasting plasma glucose, postprandial plasma glucose, and HbA1c have been measured.

The BMI used to divide the subjects studied into underweight (< 18), normal (18- < 25), overweight (25- < 30) and obese (30 and above). Normal WHR range for males =  < 0.9 and females =  < 0.85^11^.

Patients with HbA1c levels between 4.4% and 6.4% considered to be a good control group, those with HbA1c levels between 6.5% and 8.4% considered to be a moderate control group, and those with HbA1c levels 8.5% or higher rated as a poor control group^12^.

Blood pressure (BP) was measured twice using a mercury sphygmomanometer while the subject in a sitting position and the mean of the two readings was used for further analysis.

### Cardiac autonomic functions

Cardiac autonomic functions were tested by 4 standard Ewing non-invasive tests^13^ using multi-parameter electrocardiography (ECG) patient monitor (S/N 60,107,112,602) manufactured by Bestmed Technology Limited (China). Patients were asked to fast for 12 h before the procedure and to avoid taking antidepressants, neuroleptics, caffeine, nicotine, antihistamines, smoking, or alcohol. The tests were conducted between 7:00 a.m. and 9:00 a.m. in a quiet environment with a steady temperature of between 22–24° C. The tests are:

1. Autonomous parasympathetic function tests for cardiovascular control, including the following:

(a) Heart rate (HR) response to a deep breathing test:

While the subject was in a supine position with all ECG leads attached, they were asked to breathe normally for 2 min, and then asked to perform a maximum of 6 deep breaths in 1 min. Exhalation: inspiration (E: I) ratio was obtained using the following formula:

E: I ratio = mean value of the longest R-R interval during the expiration/mean value of the shortest R-R interval during inspiration.

(b) Immediate heart rate (HR) response to standing: During ECG recording, the subject was asked to lie quietly for 3 min, then asked to stand up and remain motionless (a point was identified on the ECG paper to indicate the standpoint). The 30:15 ratio was calculated by taking the ratio of the R-R interval at the 30th beat and 15th beat after standing.

2. Sympathetic autonomic function tests for adrenergic vascular regulation including the following:

(a) Blood pressure (BP) response to standing:

Patient BP was measured while they were lying down quietly and again when they stood up 1–2 min after standing. The postural decrease in BP after 2 min was taken as the difference between BP (systolic and diastolic BP) lying and the BP (systolic and diastolic BP) standing.

(b) BP response to sustained handgrip testing (BP response to static exercise):

The subject was asked to apply handgrip pressure for 1 min at 30% of the maximum voluntary contraction and, at the same time, changes in BP were observed using a sphygmomanometer. The difference between the diastolic BP just before the contraction was released, and the handgrip started was taken as a measure of the response.

Variation in HR during deep breathing (E: I ratio) was considered normal if it was ≥ 1.21 and abnormal if it was ≤ 1.10; the value of immediate HR response to standing was considered normal when it was ≥ 1.04 and abnormal when it was ≤ 1.00; the value of BP response to standing was considered normal at ≤ 10 and abnormal at ≥ 30, and the value of BP response to sustained handgrip was considered normal at ≥ 16 and abnormal at ≤ 10^4^. Each of these 4 tests was assigned a score of 0 for normal, 0.5 for borderline, and 1 for abnormal results, and the sum of these 4 tests was the Ewing score used to assess the severity of CAN^14^. To look for a sympathetic or parasympathetic condition, only abnormal and normal values were taken plus borderline cases were considered normal. Therefore, subjects with 2 abnormal HR-based tests were considered having parasympathetic neuropathy. In the case of sympathetic neuropathy, at least one abnormal test result of two BP-based tests was used to denote sympathetic neuropathy^4,15^.

### Statistical analysis

The Social Sciences Statistical Package (SPSS, version 25) was used for statistical analysis. Continuous variables were tested for normality (Shapiro Wilk test) and were found to be normally distributed. They were therefore expressed as mean ± SD and analyzed with an independent t-test or variance analysis (ANOVA). These variables were expressed as numbers and frequency and analyzed with Chi-square. The association of each variable with the development of CAN was analyzed by calculating the odds ratio (OR) with its corresponding 95% confidence interval (CI) using univariate and multivariate logistic regression. A p-value of < 0.05 was considered statistically significant.

### Ethics approval

The study was performed under the declaration of Helsinki (2008) and the Dutch Medical Research involving Human Subjects Act (WMO).

### Ethics statement

Al-Nahrain University -Baghdad, Iraq. The study approved by the Institutional Review Board (IBR) of the College of Medicine, Al-Nahrain University (Decision number: mmm/155: Date: 11/12/2018). The consent obtained in written.

## Results

### Characteristics of the studied population

Table 1 shows no significant differences in age, weight, height, BMI, WHR, and current smoking between people with diabetes and the control group. The overall values of the autonomic cardiovascular tests and the Ewing scores were different between the two groups studied. HR response to deep breathing (E: I ratio), HR response to standing up (30:15 ratio), and sustained handgrip (mmHg) was significantly decreased in people with diabetes compared to control subjects (p < 0.001). On the other hand, BP’s response to standing (mmHg) and Ewing scores was significantly higher in people with diabetes compared to control subjects (p < 0.001).

**Table 1:** Baseline characteristics of the study population.

Variable	Controls n = 100	Diabetic patients n = 142	*p*-value
**Age (years)**	51.73 ± 8.81	53.35 ± 7.83	0.109†
**Gender**			
Male	39 (39%)	62 (43.66%)	0.469‡
Female	61 (61%)	80 (56.34%)	
**Weight (Kg)**	82.85 ± 10.28	84.16 ± 10.64	0.061†
**Height (cm)**	167.94 ± 14.09	170.6 ± 12.85	0.116†
**Body mass index (kg/m**^**2**^**)**	29.16 ± 4.6	29.68 ± 3.78	0.334†
**Waist/hip ratio**	0.93 ± 0.128	0.92 ± 0.074	0.093†
**Current smoking**			
Yes	18 (18%)	34 (23.94%)	0.268‡
No	82 (82%)	108 (76.06%)	
**CAN tests**			
HR response (E: I ratio)	1.246 ± 0.065	1.129 ± 0.135	< 0.001†
HR response (30:15 ratio)	1.094 ± 0.036	1.024 ± 0.10	< 0.001†
BP response to standing (mmHg)	7.2 ± 2.5	13.345 ± 11.3	< 0.001†
BP response to sustained handgrip (mmHg)	19.64 ± 1.05	16.556 ± 4.73	< 0.001†
Ewing score	0.075 ± 0.239	1.359 ± 1.467	< 0.001†

DM = diabetes mellitus; HR = heart rate; E: I = expiration: inspiration; BP = blood pressure, CAN = cardiac autonomic neuropathy; † t-test, ‡ chi-square.

### The cardiovascular autonomic tests

Cardiovascular autonomic tests for the two groups studied were presented in Table 2. An abnormal HR response to deep breathing (E: I ratio) was found in 50% of patients versus only 4% of healthy subjects (*p* < 0.001), abnormal HR response when standing up (30:15 ratio) in 42.25% of patients versus 1% of the healthy subject (*p* < 0.001), abnormal orthostatic hypotension in 24.65% of patients versus none (0%) of the healthy subjects (*p* < 0.001), and finally, abnormal response to sustained handgrip in 14.79% patients versus none (0%) of the healthy subjects (*p* < 0.001).

**Table 2:** Cardiovascular autonomic tests of the study population.

Test		Controls n = 100	Diabetic patients n = 142	*p*-value
HR response (E: I ratio)	Abnormal Normal	4 (4%) 96 (96%)	71 (50%) 71 (50%)	< 0.001‡
HR response (30:15 ratio)	Abnormal Normal	1 (1%) 99 (99%)	60 (42.25%) 82 (55.75%)	< 0.001‡
BP response to standing	Abnormal Normal	0 (0%) 100 (100%)	35 (24.65%) 107 (75.35%)	< 0.001‡
Sustained handgrip	Abnormal Normal	0 (0%) 100 (100%)	21 (14.79%) 121 (85.21%)	< 0.001‡

DM = diabetes mellitus; HR = heart rate; E: I = expiration: inspiration; BP = blood pressure, ‡ chi-square.

### Patient’s clinical data

People with diabetes were classified according to the cardiac autonomic tests into those with CAN and comprised 75 (52.82%) patients and 67 (47.18%) patients without CAN. Eighty (56.3%) of the patients had DM for ≤ 5 years, 31 (21.83%) for 6–10 years (21.83%), and 31 (21.83%) for > 10 years (median = 6 years, interquartile range = 8 years). The study also divided the patients into three subgroups based on the glycemic control level; five (3.52%) patients had HbA1c < 6.4%, 69 (48.59%) had HbA1c between 6.5% and 8.4%, and 68 (47.8%) had HbA1c > 8.4%.

There was no evidence of a difference in age, HbA1c, BMI, and WHR between the two groups in general. However, people with diabetes with CAN have a longer duration of illness than those without CAN (*p* < 0.001). Concerning the frequency of CAN in terms of diabetic control, it was evident that with poor diabetic control, the frequency of CAN increases substantially to reach its maximum with HbA1c ≥ 8.5% with a significant difference (*p* = 0.006).

Furthermore, given the frequency of CAN in terms of duration of diabetes, the number of people with diabetes is almost doubled when the duration was 6–10 years and > 10 years compared to < 5 years (Table 3).

**Table 3:** Demographic and Ewing’s tests values of diabetic patients with and without CAN.

Variables	Diabetic patients n = 142		*p-*value
	With CAN n = 75	Without CAN n = 67	
**Age, years**	53.79 ± 6.66	53.43 ± 6.12	0.882†
**Gender**			
Females	42 (56%)	38 (56.72%)	0.932‡
Males	33 (44%)	29 (43.28%)	
**Duration of DM, years**	8.95 ± 4.19	1.544 ± 1.01	< 0.001†
≤ 5	14 (18.67%)	66 (98.51%)	< 0.001‡
6–10	30 (40%)	1 (1.49%)	< 0.001‡
> 10	31 (43.33%)	0 (0%)	< 0.001‡
**HbA1c (%)**	9.025 ± 1.37	8.10 ± 1.13	0.141†
4.4–6.4%	0 (0%)	5 (7.46%)	0.013‡
6.5–8.4%	30 (40%)	39 (58.21%)	0.01‡
≥ 8.5%	45 (60%)	23 (34.33%)	0.006‡
**BMI (Kg/m**^**2**^**)**	29.70 ± 2.77	29.75 ± 4.68	0.936†
**WHR**	0.93 ± 0.07	0.89 ± 0.06	0.162†
**HR response (E:I ratio)**	1.014 ± 0.087	1.247 ± 0.035	< 0.001†
**HR response (30:15 ratio)**	0.962 ± 0.089	1.094 ± 0.055	< 0.001†
**BP response to standing (mmHg)**	19.13 ± 12.9	6.865 ± 2.436	< 0.001†
**BP response to sustained handgrip (mmHg)**	15.027 ± 5.845	18.286 ± 1.943	< 0.001†
**Ewing score**	2.493 ± 1.143	0.089 ± 0.193	< 0.001†

CAN = cardiac autonomic neuropathy; BMI = body mass index; WHR = waist/hip ratio; HR = heart rate; E: I = expiration: inspiration; BP = blood pressure, † t-test; ‡ chi-square.

Table 3 also shows significantly higher HR response to deep breathing (E: I ratio), HR response to standing (30:15 ratio), and sustained handgrip (mmHg) in those without CAN as compared to those with CAN (p < 0.001). On the other hand, the BP response to standing (mmHg) and Ewing score was significantly higher in people with diabetes with CAN as compared to those without CAN (*p* < 0.001).

Based on positive Ewing’s tests, parasympathetic neuropathy (criterion: two abnormal functions) was seen in 56 (39.44%) cases, while sympathetic neuropathy (criterion: abnormal result in one of the sympathetic function tests) was detected in 36 (25.35%) people with diabetes.

### CAN staging

**Figure 1:**
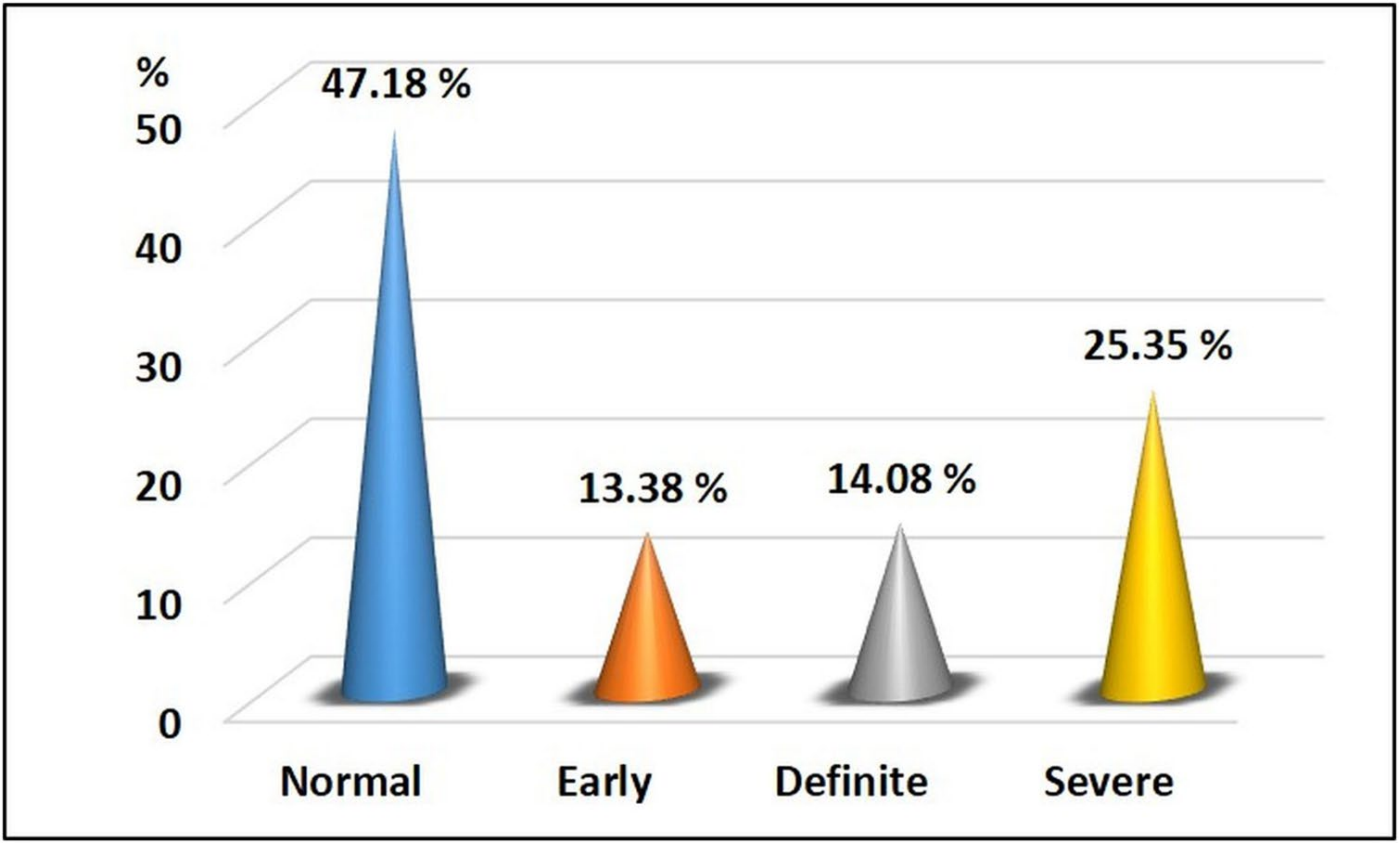
Staging of cardiac autonomic neuropathy in the diabetic patient.

Cardiac autonomic neuropathy was categorized into four different stages according to Ewing's criteria^14^: Normal = when all the cardiovascular autonomic tests were negative and consisted of 67 patients (47.18%); Early = when one of the three HR tests was abnormal and consisted of 19 patients (13.38%); Definite = when two HR tests were abnormal and included 20 (14.08%); and Severe = when two HR tests and one or both BP tests were abnormal and consisted 36 (25.35%) as shown in Fig. 1.

The characteristics of the seventy-five people with diabetes when classified according to CAN staging were presented in Table 4. Patient age and WHR within the severe group were significantly different from those in the early and definite groups (*p* = 0.028 and *p* = 0.04 respectively); while there was no significant difference (*p* = 0.422) in the BMI within the three subgroups.

**Table 4:** Characteristic of diabetic patients with CAN (using ANOVA test).

Characteristic	CAN staging n = 75			P-Value
	Early n = 19	Definite n = 20	Severe n = 36	
**Age (years)**	51.13 ± 6.72^a^	53.2 ± 5.3^a^	59.28 ± 5.14^b^	0.028†
**Gender**				
Male	8 (42.1%)	13 (65%)	12 (33.33%)	0.072
Female	11 (57.9%)	7 (35%)	24 (66.67%)	
**BMI (Kg/m**^**2**^**)**	28.63 ± 3.56	28.9 ± 2.12	30.89 ± 2.54	0.422
**WHR**	0.90 ± 0.07^a^	0.92 ± 0.07^a^	0.96 ± 0.06^b^	0.04
**Mean HbA1c %**				
4.4–6.4%	0 (0%)	0 (0%)	0 (0%)	0.122
6.5–8.4%	10 (52.63%)	15 (75%)	5 (13.89%)	< 0.001‡
≥ 8.5%	9 (47.37%)	5 (25%)	31 (86.11%)	< 0.001‡
**DM duration (years)**				
< 5	14 (73.68%)	4 (20%)	0 (0%)	< 0.001‡
6–10	5 (26.32%)	12 (60%)	13 (36.11%)	< 0.001‡
> 10	0 (0%)	4 (20%)	23 (63.89%)	< 0.001‡

CAN: cardiac autonomic neuropathy; BMI: body mass index; WHR: waist-hip ratio; DML diabetes mellitus. † t-test, ‡ chi-square. Different small letters indicate significant differences.

According to glycemic control, none of the patients have normal HbA1c at any stage of CAN. For the moderate glycemic control group, 52.63% of the people with diabetes had early CAN, 75% have definite CAN, while only 13.89% have severe CAN. For the poor glycemic control group, 47.37% of patients had early CAN, 25% had definite CAN and 86.11% of patients had severe CAN (*p* = 0.001).

In terms of duration of illness, for those of < 5 years duration, 73.68% have an early CAN, 20% have definite CAN and none have severe CAN. Considering the 6–10 years duration group, 26.32% had early CAN, 60% had definite CAN and 36.11% had severe CAN. In people with diabetes with > 10 duration of illness, none of them had early CAN, 20% had definite CAN, and 63.89% had severe CAN (*p* = 0.001).

Table 5 showed the values of different Ewing's tests in people with diabetes according to the stage of CAN. It was obvious that there was a significant difference between the four stages of CAN. Moreover, the Ewing score was significantly increased as CAN staging become more severe.

**Table 5:** Ewing’s tests values of diabetic patients (using ANOVA test).

Cardiovascular autonomic test	Diabetics patients n = 142				*p*-value
	Normal n = 67	Early n = 19	Definite n = 20	Severe n = 36	
HR response (E: I ratio)	1.247 ± 0.347^a^	1.065 ± 0.115^b^	1.005 ± 0.06^c^	0.975 ± 0.072^c^	< 0.001
HR response (30:15 ratio)	1.095 ± 0.055^a^	1.053 ± 0.079^b^	0.952 ± 0.068^c^	0.927 ± 0.067^c^	< 0.001
BP response to standing (mmHg)	6.865 ± 2.436^a^	7.633 ± 2.565^a^	7.25 ± 2.552^a^	31.81 ± 5.231^b^	< 0.001
BP response to sustained handgrip (mmHg)	18.269 ± 1.943^a^	18.526 ± 1.837^a^	18.150 ± 1.814^a^	11.444 ± 6.579^b^	< 0.001
Ewing score	0.089 ± 0.193^a^	1.0 ± 0.0^b^	2.0 ± 0.0^c^	3.556 ± 0.504^d^	< 0.001

CAN = cardiac autonomic neuropathy; HR = heart rate; E: I = expiration: inspiration; BP = blood pressure, Different small letters indicate significant differences.

### CAN severity, demographic & clinical data

**Figure 2:**
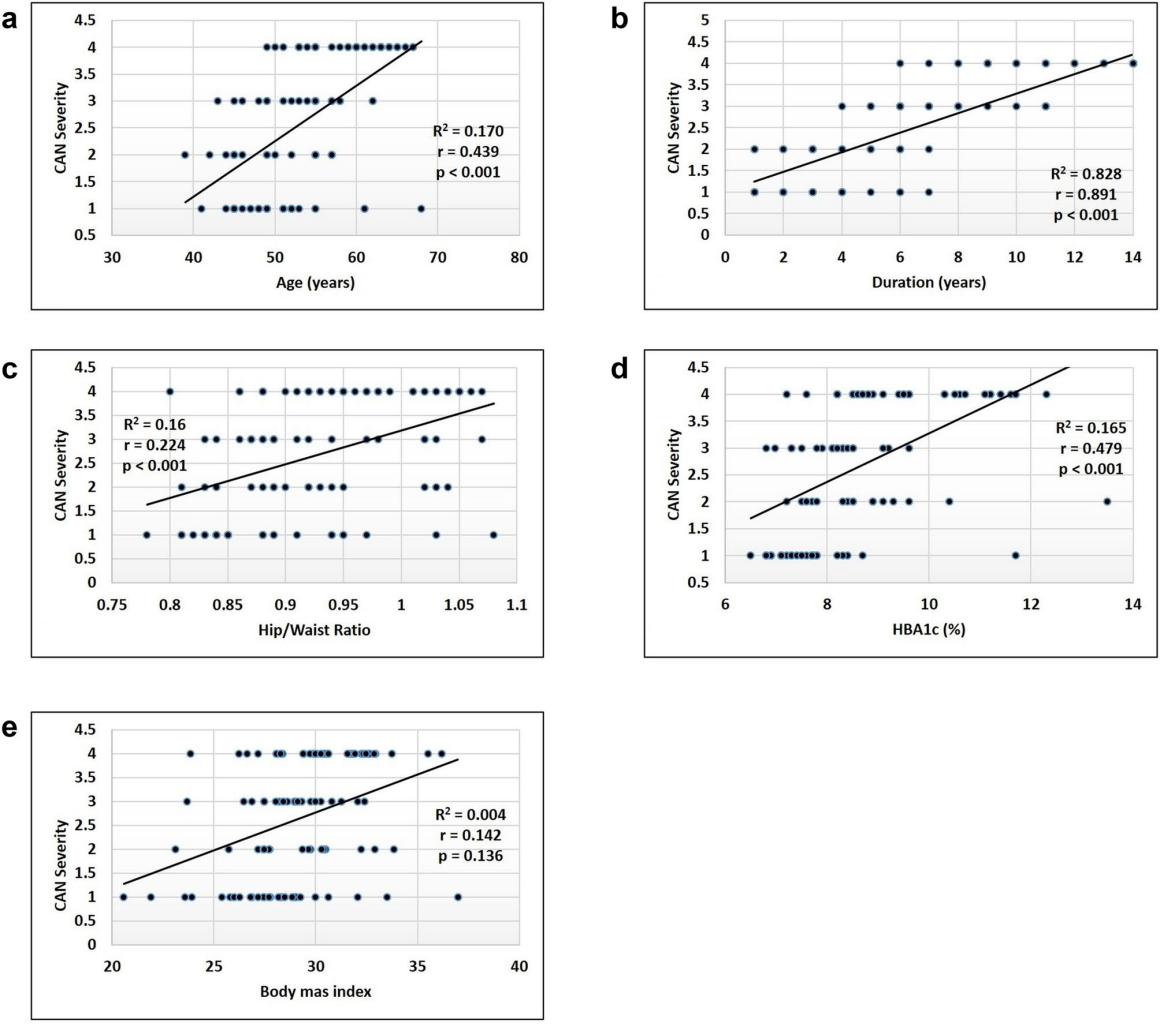
(**A**) correlation between CAN severity and patient’s age (**B**) correlation between CAN severity and diabetes duration (**C**) correlation between CAN severity and waist-hip ratio (**D**) Correlation between CAN severity and HbA1c level (**E**) Correlation between CAN severity and body mass index.

Using Pearson's correlation test, Fig. 2 (A-E) shows a positive correlation between CAN severity and patient age (r = 0.439, *p* < 0.001), duration of diabetes (r = 0.891, *p* < 0.001), WHR (r = 0.224, *p* < 0.001), HbA1c (r = 0.479, *p* < 0.001), and WHR (r = 0.224, *p* < 0.001). On the contrary, there was no significant correlation between the CAN severity and the BMI (r = 0.142, *p* > 0.136).

### Association of CAN with demographic features of people with diabetes

Of the seven characteristics of people with diabetes, three were found to be significantly associated with CAN when tested separately. These were the age, duration of diabetes, and HbA1c level. However, using multivariate regression analysis is used to test all characteristics together; only the duration of diabetes and HbA1c level appear to be associated with CAN development (Table 6).

**Table 6:** Predictors of cardiac autonomic neuropathy (using multiple regression).

Variables	CAN		Crude p-value	Adjusted p-value	OR (95%CI)
	Positive (n = 75)	Negative (n = 67)			
**Age (years)**					
< 50	16 (21.33%)	31 (46.26%)	0.002	0.154	1.0 Reference
≥ 50	59 (78.67%)	36 (53.7%)			2.21 (0.74–6.57)
**Gender**					
Male	33 (44%)	29 (43.28%)	0.932	0.409	1.0 Reference
Female	42 (56%)	38 (56.72%)			0.63 (0.21–1.89)
**BMI (Kg/m**^**2**^**)**					
< 30	41 (54.67%)	41 (84%)	0.432	0.492	1.0 Reference
≥ 30	34 (45.33%)	26 (16%)			1.56 (0.44–5.57)
**WHR**					
< 0.9	27 (36%)	32 (68%)	0.156	0.575	1.0 Reference
≥ 0.9	48 (64%)	35 (32%)			0.58 (0.09–3.93)
**Smoking**					
**Yes**	22 (29.33%)	12 (16%)	0.111	0.318	1.0 Reference
**No**	53 (70.67%)	55 (84%)			0.94 (0.12–7.28)
**Duration (years)**					
< 7.5	32 (42.67%)	62 (100%)	< 0.001	0.001	1.0 Reference
≥ 7.5	43 (57.33%)	5 (0%)			10.63 (2.56–44.06)
**HbA1c**%					
< 8.0	16 (21.33%)	54 (84%)	< 0.001	0.008	1.0 Reference
≥ 8.0	59 (78.67%)	13 (16%)			4.25 (1.45–12.43)

CAN = cardiac autonomic neuropathy; BMI = body mass index; WHR = waist-hip ratio.

Approximately 57% of patients with diabetes duration ≥ 7.5 years were CAN positive compared to only 42.67% of patients with duration < 7.5 years and CAN positive (OR = 10.63, 95% Cl = 2.56 – 44.06). Likewise, the majority of patients (78.67%) with HbA1c ≥ 8.0 developed CAN compared to only about one-fourth of those with HbA1c < 8.0 who developed this a complication (OR = 4.25, 95% Cl = 1.45–12.43).

## Discussion

### The study population

#### Cardiovascular autonomic tests

People with diabetes show significantly abnormal cardiac autonomic tests (from 14.79 to 50%) compared to 0 to 4% of control subjects. Other researchers^14,15^ record similar results, but with slightly different percentages that could be due to the CAN adoption criteria, patient sample size, disease length, and ethnicity.

This tendency of people with diabetes to develop CAN is attributed to complex interactions between several mechanisms and pathways leading to neuronal ischemia and finally neuronal death^16,17^.

Hyperglycemia is the leading cause of diabetes-related CAN. This induces oxidative stress and toxic advanced glycosylation products which ultimately result in neuronal dysfunction and increases mitochondrial production of free reactive oxygen species, causing oxidative damage to the microvasculature that supplies these peripheral nerves. These different pathways induce changes in gene expression, transcription factors, interruption of several cell functions, and communication between the cells and the surrounding matrix. All this leads to neuronal dysfunction and death^16,18^.

Like somatic neuropathies, some investigators have shown that autonomic nerves may be affected in a length-dependent manner^17^. As a result, CAN often first manifests itself in the vagus nerve, the longest parasympathetic autonomic nerve in the body, and the one responsible for nearly three-quarters of parasympathetic activity. Compassionate denervation occurs at the later stage of CAN^19^.

People with diabetes in this cohort showed significantly reduced HR response to deep breathing (E:I ratio), HR response to standing (30:15 ratio), BP response to handgrip testing, and orthostatic hypotension. These findings have also been demonstrated by others^20–22^.

### The people with diabetes

#### The clinical data

In our study, approximately 53% of the people with diabetes presented with CAN, were within the range (15.3% to 61.6%) registered worldwide^23–25^. This wide range is due to the variability of diagnostic criteria, and tests used among T2DM patients.

Our study revealed a non-significant difference in the mean age of the diabetics with and without CAN which denotes no relationship between CAN and age^25,26^. Other groups of researchers have seen almost the same age incidence^25,27^.

Although not statistically significant, women had a higher prevalence of CAN than men in this study (56% vs. 44%). Approximately similar results have been reported by others^25,28^. The gender impacts on CAN epidemiology is controversial. In the Action to Control Cardiovascular Risk in Diabetes (ACCORD) study, which included > 8,000 patients with T2DM, a higher prevalence of CAN was found in women compared to men^19^. A more recent study, although not statistically significant, also showed that women had a higher prevalence of CAN than men (65.2% vs. 34.8%, p = 0.059)^29^. However, in a multi-center, cross-sectional study of 3,250 people with diabetes, the prevalence of CAN was not different between males and males (35% male vs. 37% female)^30^. Other studies showed no difference in CAN prevalence between men and women^26,31,32^.

In our study, the prevalence of CAN increases as the duration of diabetes increases (beyond 6 years and more) and also with poorer glycemic control (6.5–8.4% and > 8.4%). In their studies, Pop-Busui^19^, Spallone et al.^7^, Moningi et al.^33^, and Bhuyan et al.^34^ have shown that the longer duration of diabetes is independently associated with CAN. Ahire et al.^35^ found a positive correlation between CAN and the duration of diabetes, as those with a duration of > 5 years are more likely to have a definite and severe CAN than those with a duration of < 5 years. Also, early CAN was commonly seen in patients with a shorter duration of disease. The incidence of CAN was reported to be 2% annually in patients with T2DM^16^ and the prevalence increased from 9 to 31% 1 year later^23^. In addition, Dimova et al.^36^ found that prevalence increased from 19.8% in pre-diabetics to 32.2% in newly diagnosed patients with T2DM, with the higher prevalence reported in patients with T2DM and longer duration of disease. The increased prevalence of CAN with a longer duration of the disease can be attributed to the fact that patients often have a history of many years without symptoms during which blood glucose peaks occur unnoticed, but diabetes is not yet diagnosed and treated. Thus, in T2DM, complications of diabetes may occur at the time of the initial presentation.

#### CAN staging

Compared to the patients with early or definite CAN, patients with severe CAN were older at the time of the study (p = 0.028), had longer diabetes duration, and poorer glycemic control than HbA1c indexed. Yun et al.^37^ study the progression of CAN and its association with other risk factors in 578 people with diabetes. They noted a significant association with age, glycemic control, and disease duration. Generally, "aging of regulatory systems" in people with diabetes with CAN is 10 years earlier than the biological age of the patient^38^. This confirms the hypothesis that the aging of the autonomic system in addition to endothelial dysfunction is one of the most important reasons for the early onset of atherosclerosis and associated cardiovascular disease. Yun et al.^39^ and Lai et al.^40^ examined diabetics with CAN and observed delayed, sympathetic and parasympathetic activation in response to fluctuations in blood glucose levels. This study showed a step-by-step increase in the risk of developing higher HbA1c variability based on CAN severity, suggesting a dose-dependent negative CAN influence. The complementary interaction of the hormones and peptides involved in glucose homeostasis is usually more disrupted as diabetes progresses, and subjects are likely to show a high increase in glucose levels from external stimuli. The effect of CAN on glucose fluctuations could, therefore be more pronounced in subjects with long-term diabetes^41^.

#### Cardiovascular autonomic tests

In general, there was a significant difference between people with diabetes with and without CAN in the results of the Ewing tests. In line with this finding, Lin et al.^21^ examined 90 people with diabetes with and without CAN and found the same difference.

Autonomous innervation is the primary extrinsic control mechanism that regulates HRV and cardiac performance. Chronic hyperglycemia has been shown to promote progressive autonomic neural dysfunction in a manner that parallels the development of peripheral neuropathy^16,19^.

The criterion for CAN severity was an increase of at least one point in the Spallone score. The E/I ratio, the 30:15 ratio, and the sustained handgrip were significantly reduced while the BP response to standing was increased. These cardiovascular autonomic changes are expected to have a longer duration of diabetes due to the impact of CAN severity^42^.

This study found a significant correlation between CAN and glycemic control, duration of diabetes, and WHR, but not BMI. These findings underscore the role of insulin resistance not only in the etiology of metabolic syndrome but also as a determinant of cardiac autonomic dysfunction. These findings are consistent with the reports by Laitinen et al.^43^, and Moţăţăianu et al.^44^.

Absent BMI and CAN correlations may be explained by the fact that BMI quantifies general adiposity; although individuals who are overweight or obese are likely to have excess fat, BMI does not indicate how this fat is distributed in the body. Welborn and Dhaliwal^45^ and Srikanthan et al.^46^ have confirmed that WHR is a superior clinical measure for predicting all-cause and cardiovascular disease mortality.

Contrary to our study, no relationship was found in a recent study in Iran between the CAN and the glycemic control level^47^. They argued that this could be due to several reasons that could affect outcomes such as criteria for tight control of diabetes, duration of diabetes, metabolic memory, sample size, and the CAN diagnostic method.

Of interest based on positive Ewing tests, we found that the parameters used to evaluate parasympathetic functions were abnormal (positive) in 56 (39.44%) compared to 36 (25.35%). It may indicate that parasympathetic dysfunction is more sensitive to the detection of autonomic dysfunction. These percentages were slightly different from those registered worldwide, yet they were harmonized^14,21^.

### Effect of demographic features on the development of CAN

With multivariate regression analysis to test age, gender, BMI, HWR, smoking, HbA1c level, and disease duration, only the duration of diabetes and HbA1c level appear to be associated with CAN development. This is partly in agreement with the findings of others^7,29^ who used clinical and laboratory predictors.

## Conclusion

CAN is common among people with diabetes (52.82%), the poorer the glycemic control and the longer the duration of the disease, the higher the incidence of CAN in T2DM. Age, duration of disease, WHR, and HbA1c are well correlated with the severity of CAN. Parasympathetic impairment is more sensitive to the detection of autonomic dysfunction.

CAN is a significant DM complication and is closely associated with increased risk of cardiovascular mortality. While it is a popular complication, its importance has not been fully understood in our societies and still hot field for research because: (A) The impact of sex on CAN epidemiology is controversial. (B) The effect of ethnicity on CAN prevalence is controversial and little to nothing has been done in Arabs to the best of our knowledge.

(C) CAN pathogenesis is complex, multifactorial, and still under much debate. (D) Smoking, obesity, and lifestyle are among the risk factors and were certainly different between societies (Arab versus western countries)”.

## Data Availability

All relevant data are within the manuscript and its Supporting Information files.

## Data availability

The data that support the findings of this study are available from the corresponding author upon reasonable request.
